# Smoking-Related Social Control in Indonesian Single-Smoker Couples

**DOI:** 10.1007/s12529-020-09935-z

**Published:** 2020-11-10

**Authors:** DA Ayuningtyas, Marrit Tuinman, Yayi Suryo Prabandari, Mariët Hagedoorn

**Affiliations:** 1 Department of Health Psychology, University of Groningen, University Medical Center Groningen, Groningen, Netherlands; 2 Department of Health Behavior, Environment Health and Social Medicine, Faculty of Medicine, Public Health and Nursing, Universitas Gadjah Mada, Yogyakarta, Indonesia

**Keywords:** Smoking, Spouses, Qualitative research, Social control, Health behaviour

## Abstract

**Background:**

The majority of Indonesian smokers are men and those who are married nearly always have a non-smoking wife (i.e. single-smoker couples). Previous studies have suggested that Indonesian women dislike smoking. However, contesting their husbands’ smoking could be seen as disrespectful. In this study, we examine whether, and if so how, wives employ social control tactics to change their husbands’ smoking and how the smokers perceive the tactics.

**Method:**

In-depth interviews (*N* = 12) with five single-smoker couples (*N* = 10 individual interviews) and two non-smoking wives of smokers (*N* = 2) were conducted in Jogjakarta, Indonesia. We used a social control framework and thematic analysis approach to analyse the transcribed interviews.

**Results:**

Three themes emerged from smokers and their wives: (1) although the wives know that smoking is bad, they have to tolerate it, (2) wives and their husbands find it important to maintain harmony and (3) their family’s needs serve as common ground. All the wives interviewed exerted social control to some degree, especially when they were pregnant or had children. Smokers reacted positively to social control and agreed to child-related house rules, but not to requests to give up smoking.

**Conclusion:**

Wives do exert social control and smokers are willing to accommodate and adapt their smoking. However, wives’ influence on smoking may be limited in Indonesia, and focusing on managing their husbands’ smoking at home rather than overall smoking might be more fruitful.

**Electronic supplementary material:**

The online version of this article (10.1007/s12529-020-09935-z) contains supplementary material, which is available to authorized users.

## Introduction

Indonesia has the highest prevalence of male smokers in the world, where 62.9% of men smoke daily, compared with only 4.8% of women [1]. While an overall decrease in smoking is taking place across the world, most countries will not achieve the WHO’s target of a 30% reduction in tobacco use by 2025 [2]. Smoking rates are even expected to increase in some developing countries, such as Burkina Faso, Pakistan and Indonesia [3]. In 2010, 31% of men smoked in Burkina Faso and 38% in Pakistan, and this is expected to increase to 49% and 45% respectively by 2025 [3]. The projection for Indonesia is even higher, at 87.2% for men in 2025 [3].

Indonesia has a long history of growing and trading tobacco [4], which has led to smoking being extensively linked to cultural practices. Smoking in Indonesia serves many purposes, from socialising and signifying maturity and masculinity to emotion regulation [5]. However, smoking is generally only acceptable for men, and there is strong cultural disapproval of women smoking [4]. Although it is more acceptable for ‘modern’ women in urban areas to smoke [4, 5], female smokers in Indonesia risk being considered ‘bad girls’ or ill-mannered [6].

Considering the proportion of male and female smokers in Indonesia [7], the majority of married male smokers have non-smoking wives, with whom they form so-called single-smoker couples [8]. Research among Western smokers has shown that having a non-smoking spouse is associated with a higher intention to stop smoking [9] and a greater chance of actually stopping [10]. Having a non-smoking spouse may influence smoking behaviour because non-smoking spouses are more supportive of attempts to stop than smoking spouses are [11]. There have also been suggestions that non-smoking spouses are likely to try to change their spouses’ smoking behaviour [12, 13]. This is referred to as social control, which is defined as an interaction that involves explicit attempts to regulate, influence and constrain the other’s behaviour [14]. Social control has been proposed as one of the mechanisms that could explain the benefits of marriage on health [15].

However, the positive association between having a non-smoking spouse and stopping smoking does not seem to apply to Indonesian and other Asian smokers. In countries such as Bangladesh, China and Timor-Leste, the ratio of male to female smokers reaches over 10:1 [16, 17], suggesting that single-smoker couples are also common in these countries. However, studies among Bangladeshi and Saudi smokers have shown that being married does not predict smoking cessation [18, 19]. Studies among Chinese smokers have suggested that non-smoking wives have a limited influence on smoking cessation and report that smokers ignore their wives’ interventions to make them stop smoking [20]. While the smokers accepted smoking restriction rules when their wives were pregnant [12], only a quarter quit smoking [21].

Despite the high number of smokers and the high acceptability of smoking among Indonesian men, many Indonesian women do not have a favourable view of smoking and report preferring a partner who does not smoke [22]. As women also function as carers of their families’ health [4], it is reasonable to think that they would try to challenge or change their husbands’ smoking behaviour. There is a lack of attention to wives and other social factors in Indonesian smoking studies. However, one study reported that the majority of wives disapprove of their husbands’ smoking inside the house and ask them not to do so [23]. Unfortunately, their requests are largely ignored, and the wives reported being unable to change their husbands’ smoking behaviour. While the findings showed that Indonesian men ignored their wives’ direct requests [23], they might respond differently to other social control tactics that were not explored in the study.

Social control can take the form of various tactics, which are classified as positive or negative tactics [24]. Positive tactics include behaviours such as bargaining or using humour, while negative tactics include displaying negative emotions or attempting to induce these in the target, for example by nagging or withdrawing affection [24]. Findings on the effectiveness of social control with regard to health have so far been mixed. Some studies found social control to be effective for changing various health behaviours, such as increasing physical activity [25], losing weight [26] and cutting back on smoking [27], while others found it to be ineffective. Social control can also backfire, increasing health-compromising behaviours such as smoking and causing greater psychological distress [28] or decreasing healthy behaviours such as physical activity [29] and dietary adherence [30]. In their meta-analysis, Craddock et al. stressed the importance of distinguishing between types of social control tactics, as they have different effects on the target behaviour [31]. It is suggested that negative tactics are likely to backfire, while positive tactics were found to have the desired effect on health behaviours [31].

It is essential to examine the context in which social control is exerted, as this determines how it will be received [31]. Gender is one of the context variables to consider as, in marital relationships, men are more likely to be the recipient of social control than women [32]. However, previous studies that examined how a recipient’s gender influenced how they reacted to their spouse’s social control produced mixed results. Some found that a positive behavioural change was observed in men [27, 33, 34], while others found the opposite [28, 35, 36]. Unfortunately, some of these studies did not distinguish between positive and negative tactics [27, 28]. Nevertheless, the ones that did also reported mixed results in how men react to social control. There is also a concern that spousal social control undermines men’s ability to take care of themselves [28] or that it threatens men’s freedom or masculinity [36].

Culture also contributes to the context of social control. It has been suggested that, in several Asian countries such as Indonesia, India and China, a woman trying to persuade her husband or father-in-law to stop smoking could be seen as disrespectful [5, 37]. However, this does not mean that Asian women are powerless. A study in China showed how women managed their husbands’ smoking at home through confrontation with their husbands, allying with their mother-in-law or subtly persuading their father-in-law [38]. Although they could not control their husbands’ overall smoking patterns, they had enough power to control smoking in their private space [37]. The tactics that these women used, such as allying with their mother-in-law [38], were culture-specific and adapted to their position and power, something that has not received much attention in Western spousal social control studies.

There have been very few studies in spousal social control in Asia. While a few qualitative studies have been conducted among Chinese smokers [12, 37, 38], to our knowledge, no other studies have been carried out in other Asian countries. The only Indonesian study by Nichter et al. surveyed both husbands and wives of single-smoking couples about second-hand smoking exposure in their houses [23], however, social control was not the focus of the study. Little information was obtained regarding the context and how the wives interacted with their husbands concerning smoking. It was also not known whether the wives tried different tactics. As previously mentioned, the type of tactics used could determine the effectiveness of social control. Studying the context in which spousal social control takes place and the tactics that wives employ could help to understand the role that wives can play in changing Indonesian men’s smoking behaviour. In this study, we aim to address this knowledge gap by examining how wives use social control in Indonesia and how it is perceived and received by smokers.

## Methods

### Recruitment

Participants were recruited from various neighbourhoods in the city of Jogjakarta and Sleman Regency in Indonesia. We used convenience and snowball sampling methods to recruit participants. The participants were approached through the first author’s social circle and through an online advertisement placed in July and August 2018. The first author sent the recruitment poster to her social circle in WhatsApp groups, such as a group of university friends, with the request to forward it to people they knew who might be eligible. We recruited through WhatsApp as it is one of the main communication channels used in Indonesia. The first author also posted the recruitment poster on her Facebook, Instagram and Twitter pages, again with the request to share and forward it to others. We also asked several Indonesian public and semi-public figures with sizeable follower counts on Twitter to share the poster, to reach a wider audience. Lastly, we asked the participants to recommend anyone else who fitted the inclusion criteria. Although we posted the recruitment poster on several social media channels, only one participant responded to the recruitment post on Instagram. Most of the participants were recruited through the first author’s friends (*N* = 5), and four were recruited through snowball sampling. Neither the participant recruited through Instagram nor the participants recruited through the first author’s friends had any relationship to the first author, although one of them was acquainted with the second interviewer. In this case, the interviewer was assigned to interview the participant’s wife to prevent the participant from feeling uncomfortable about taking part in the study. None of the participants recruited by snowball sampling knew any of the interviewers. Prior to the interviews, we sent the potential participants a text message to introduce the study and to ensure that they fulfilled our inclusion criteria.

The inclusion criteria were (1) the participants were part of a single-smoker relationship, either as a male smoker or a non-smoking wife, (2) the male smoker was a daily smoker, (3) the participants had been in the relationship for at least 1 year, (4) the participants cohabited, (5) both partners were aged 18 years or older and (6) neither participant suffered from smoking-related chronic illnesses such as bronchitis. We excluded illnesses that participants might associate strongly with smoking (i.e. related to the respiratory tract) as smoking-related illnesses could strongly influence the decision to stop smoking and we were explicitly interested in the wife’s influence. We recruited both couples and individual participants who met the inclusion criteria, as the data were not analysed at a dyadic level but at a group level (smokers and wives).

### Procedure

We employed a qualitative approach, using in-depth interviews to collect data for this study. The interview guide was informed by the social control framework, in which we asked questions about the wives’ use of social control to influence their husbands’ smoking behaviour. Prior to data collection, we obtained a permit from the province and municipality of Jogjakarta to conduct research and ethical approval from the Medical and Health Research Ethics Committee of Universitas Gadjah Mada, Indonesia.

The first author initially contacted every participant to briefly introduce the study. The interviews were conducted by two female interviewers (the first author and a Clinical Psychology Master’s student). Both interviewers were trained in observation and interview techniques as a part of their studies and through additional courses.

All participants consented to the interviews and to having their interviews recorded. The interviews lasted between 20 and 70 minutes and were conducted in a private or semi-private area at a time chosen by the participants. Most were conducted at the participants’ homes (seven interviews) or workplaces (four interviews) and one in a restaurant. In most interviews, only the interviewer and the participant were present at the location, with the exception of three interviews that were conducted in the participants’ workplaces and the interview conducted in a restaurant. Each interview was conducted once. The smokers and their wives were interviewed separately and given a small gift afterwards of a hand towel and a sticker to thank them for their participation. We continued the recruitment process until data saturation was reached, which was defined as eliciting no new content regarding the wives’ social control and the smokers’ responses in the interviews.

The semi-structured interviews were conducted in Indonesian, and the topics included (1) smoking behaviour, (2) how the non-smoking wives perceived smoking, (3) whether and how they tried to make their husbands stop smoking, (4) how the smokers perceived and reacted to the attempts, (5) whether the attempts had any effect on the smoking behaviour and (6) what would change the smokers’ behaviour. The interview guide is available as Electronic Supplementary Material.

### Data Analysis

All the interviews were audio-recorded and transcribed verbatim, using the thematic analysis approach to identify, analyse and report patterns or themes in the data [39]. We employed data source and investigator triangulation to achieve reliability. Data source triangulation was obtained by interviewing both smokers and their wives to obtain perspectives from both parties in the relationship. We also strived to attain investigator triangulation by having the interviews coded by the first author and a second independent coder who was not involved in the interview process.

The interviews were coded on the raw data in Indonesian using OpenCode 4.03. The codes were derived from the data, and the coding process was done independently. All coding discrepancies were discussed until full agreement was reached; then, the codes were grouped into categories to analyse the variation in the data. Finally, the themes were constructed based on the categories. Every step of the analytical process was first done in Indonesian and later translated into English by the first author to facilitate discussion with the other authors. The translation of the participants’ quotes in this article was checked by three bilingual Indonesian-English speakers who were not affiliated with the study.

## Results

### Sample Characteristics

In-depth interviews (*N* = 12) with five single-smoker couples (*N* = 10 individual interviews) and two non-smoking wives of smokers (*N* = 2) were conducted in Jogjakarta, Indonesia. Table 1 presents the characteristics of the study participants. The smokers had a mean age of 31, smoked on average 13 cigarettes a day and had been in the relationship for an average of 4 years. The mean age of the wives was 30. All participants in this study were in a heterosexual relationship and were given pseudonyms for the purpose of this article.

**Table 1:** Characteristics of the study sample

Characteristic	Smoker (n = 5) No. (%)	Non-smoking wife (n = 7) No. (%)
Sex		
Male	5 (100)	-
Female	-	7 (100)
Education		
Low	-	-
Middle	1 (20)	1 (14)
High	4 (80)	6 (86)
**Characteristic**	**Smoker (n = 5)** **Mean (SD)**	**Non-smoking wife (n = 7)** **Mean (SD)**
Age	31 (5)	30 (5)
Number of cigarettes/day	13 (7)	-
Relationship duration	4 (3)	4 (3)
Family members in the household	5 (5)	5 (4)

### Themes

The wives wanted their husbands to stop smoking, but this was not always manifested in an actual act. The themes below capture how the wives usually react to smoking and the smokers’ response to this.

**‘Smoking Is Bad, but I Have to Tolerate It.’** All the wives considered smoking bad for various reasons, including cleanliness issues, possible adverse effects on the health of the smokers and other family members and the cost of smoking. Despite their negative views of smoking, all wives said that they tolerated it to a certain extent. As one of the wives said, smoking was tolerable under certain circumstances and there was no point in asking her husband to stop if he did not intend to himself:

*I’m tired. I’m tired of complaining, I’m tired of inhaling [smoke]… Right. But he still can’t… Stop, he can’t stop yet, he said. Then… As his partner I have to be patient.* (Reika, wife).

Another wife said that, despite all the adverse effects of smoking, smoking was a right and smokers should be allowed to make their own choice:

*It’s up to the smokers if they don’t want to be healthy. I don’t have any problem with it.* (Nisa, wife).

On the other hand, the fear of jeopardising their relationship was also mentioned as a reason not to insist that the smoker stopped smoking, as well as the fact that a wife had to consider her husband’s feelings:

*He just won’t listen, what can I do? If I for example as a wife… [If I] Keep nagging… I mean I also have to think about, I mean he’s a man… You know? … I have to respect him as a man if he wants to smoke, then by all means. … I mean I would feel a bit awkward I mean he’s a man… [He’s my] husband [the] head of the family if I keep [saying] you have to do this and this and this and this! What if he… I’m afraid, if he’s not allowed to smoke then he started doing something worse I’m afraid of that.* (Rahma, wife).

Rahma said that she had to respect her husband and his decision to smoke because he is a man, but she also talked about how he often failed to honour her request for him not to smoke near her. While Rahma was the only one who explicitly discussed fear and the perceived status difference between a husband and his wife, the fact that the husband—as the head of the family—ultimately has more power than the wife was echoed by other participants. Additionally, the view that the decision to keep smoking is closely linked to masculinity and patriarchy was also mentioned by both smokers and their wives:

*My wife doesn’t really have a big effect on making me quit smoking. … Maybe because I think, ‘your wife is afraid of you’ (laughs). So I just ignore her. I have no intention [to quit] if she’s the one asking.* (Tirta, smoker).

The wives in this study tried to reconcile their negative views of smoking and their husbands’ smoking habits in different ways. Some wives tried to demand that their husbands stop smoking, but others realised that they did not have enough power to ask, and reconciled this by targeting a different behaviour, such as urging the smoker to exercise to counteract smoking’s harmful effects, or by accepting smoking as something that their husband needed or had a right to.

**Maintaining harmony: exerting and receiving social control.** The notion of being considerate of the husband’s feelings was also reflected in the use of social control by the wives. The wives employed a variety of tactics to influence their husbands’ smoking, namely enjoyable communication, negotiation, confrontation and support.

***Enjoyable Communication.*** This tactic included talking about smoking in a light-hearted manner, making jokes or by only talking about smoking at certain times. According to the wives, this prevented them from making the situation worse, as stressing the smokers could trigger them to smoke more to alleviate the stress:

*That’s also an evaluation point for me maybe I came off as nagging when I told him to quit smoking, so he got stressed, he then had more reason to smoke[,] right? For stress release.* (Alissa, wife).

***Negotiation.*** Negotiation seems to be one of the main social control tactics used by the participants in this study. For most couples, negotiation took the form of house rules, where the smokers could keep smoking as long as they followed the wives’ rules. This afforded the wives more power in decreasing second-hand smoking at home, which is inhaling other people’s cigarette smoke:

*He has to change [his clothes] and take a shower [after smoking] I don’t care. If he won’t, then sleep in another room. Sometimes he’s too tired [and says] ‘I can’t’ ‘Fine! But don’t sleep here.’* (Reika, wife).

Another common rule was to limit the places where the smokers could smoke. For example, smoking was not allowed around the wife or only allowed outside the house or at a special smoking place:

*My neighbour was pregnant and they complained about the smoke, then a few months afterwards I also got pregnant so we started to really talk about it. Apparently, it’s quite important, cigarette smoke was really bothersome. So we finally put [the smoking area] upstairs, it’s okay. No more smoke.* (Fira, wife).

The smokers in this study were relatively accepting of their wives’ attempts to change their smoking behaviour. The wives’ social control was considered normal from two standpoints: (1) that of a wife, whose role is to take care of the health of the family, and (2) that of a non-smoker, whose complaints about smoking are acceptable. Almost all of the smokers also reported not smoking when their wives were around, either by going elsewhere or by extinguishing the cigarette when their wife joined them. Most of the smokers were willing to accommodate through negotiation: obeying the smoking rules or accepting their wife’s suggestion to reduce smoking:

*[Interviewer: So [wife’s] nagging has an effect on you.] Of course. I think of it. I mean we are married. She will be my partner for life. Well… If in the beginning I already don’t listen to her what is it going to be like later? So we negotiate.* (Deni, smoker).

***Confrontation.*** Some wives chose to be more direct and demanding, by nagging, bringing up the smoker’s illness in the past or trying to scare him by talking about the harmful effects of smoking and passive smoking. They also mentioned boycotting the smokers, for example by hiding, throwing away or rationing the cigarettes, or even employing their children in the process:

*When my first daughter was little we used to live with my in-laws so I [told her] ‘if you find [cigarettes] you have to throw them away.’ (laughs) *(Alissa, wife).

Direct, confrontational tactics gained mixed views from the wives, with some reporting that they thought it would backfire:

*[My smoking friends] were all the same. They got told off [by their wives] nicely, it didn’t work. They would say yes, but they still smoked behind their wives’ back. Those who were sternly told off, they wouldn’t smoke at home. But they got even worse behind their [wives’ back]. I’m worried about it. There’s no honesty. … I don’t know whether I should be stern and risk [husband] smoking behind me or talk nicely.* (Nisa, wife).

The smokers agreed that this type of tactic would evoke rejection, for example by lying or making false promises:

*Maybe [instead of nagging] she has to wait until I want to do it, so she [should] just follow me, so I [would] say ‘okay I’ll smoke less’ if it is just [because of] her telling me to quit I will just say yes but if [she] wait[ed] until I actually wanted to, then maybe I could [quit]. So so I wouldn’t feel burdened about having to quit* (Tirta, smoker).

Here we see that Tirta rejected his wife’s request, but tried to do it in a way that ensures harmony in the relationship by lying instead of directly refusing. Another way in which the smokers rejected their wives’ attempts was by hiding their smoking or giving light-hearted and humorous excuses as to why they would not stop. The smokers also reported feeling burdened like Tirta, or guilty, especially when they fell ill and their wife had to take care of them. They also experienced guilt when someone else in the family became ill from second-hand smoking.

There was also a strongly negative view from a few smokers that, if a wife could make a smoker stop, it signified an unhealthy relationship:

*If a wife can make… [a smoker] quit smoking then we can see that she can do anything then to… the husband. As in for all decisions, it might be the wife [who was behind it]. There are some people like that but they ended up getting a divorce. So the smoking case really showed a lot of things. He indeed quit, but it showed that their relationship is bad. He didn’t smoke at home. But he smoked outside. Didn’t smoke at all at home. If he went home he had to brush his teeth and stuff. That’s not right.* (Roy, smoker).

Roy saw a dominant wife as threatening a man’s position, who after all was supposed to be the most powerful person in the household. Roy probably perceived the behaviour of the smoker in his story as a deliberate attempt to hide smoking instead of simply cleaning up and being considerate to his wife, which Roy also did as his wife requested. In Roy’s story, the wife had crossed a line by pushing the husband so far as to hide his smoking.

Certain social control tactics that breached the smokers’ autonomy to smoke, such as breaking or throwing away the cigarettes, could result in the smokers getting angry or starting a fight. All of the smokers agreed that they should be the ones to decide whether or not they would stop smoking, so any tactics that threatened their autonomy to smoke were always met with rejection. The smokers’ rejection, even when it was done quietly, would evoke a response from the wives in which their use or choice of social control tactics would be adjusted. Many wives said that they grew tired of talking about smoking as the smokers would ignore them or how the wives had to play nice or wait patiently:

*Maybe he was um really stressed or something, then I was very bothered with [his] smoking, [we talked about smoking] has happened once or twice. But I always regret it [afterwards]. Because then he hides it from me, or he doesn’t show it. Then [I find out] that [the smoking is] really bad. So I just let him be, as long as I know [about it].* (Fira, wife).

***Support.*** The wives also reported trying to alter their husband’s smoking behaviour in ways that were more supportive, for example by finding a replacement for cigarettes such as chewing gum or snacks, by suggesting alternative treatments or by motivating them in some other way to stop smoking:

*I suggested [trying] candies to him. If you can swap [cigarettes] with candies or try a new activity, and if a friend asks you to smoke maybe say no. *(Nisa, wife).

In summary, this theme describes how the wives tried to influence their husband’s smoking and how the smokers reacted to this. Most wives in this study favoured non-confrontational tactics and aimed to reduce their exposure to second-hand smoke. The smokers tended to respond to their wives’ social control by accommodating or agreeing to their requests to limit the smoking space or to cut back on smoking. Very rarely were there fights or other strong negative reactions. The smokers, like the wives, wanted to avoid confrontation: they tried to reject the social control peacefully through half-hearted promises and by making light of the situation. Because the smokers mostly accepted or quietly rejected social control, smoking was seldom reported as being a problem in the relationship. However, if the smokers chose to hide their smoking in their attempt to maintain harmony, this could backfire and cause problems.

An interesting finding in this theme was that all the wives exerted social control to some degree, even those who said that they did not mind their husbands smoking or were reluctant to make their husbands stop smoking. Those who were reluctant to ask their husbands to stop smoking would not necessarily be lenient but would apply tactics that aimed to achieve more attainable outcomes, such as reducing smoking or limiting smoking spaces.

**Family’s need as common ground.** The birth of a child and planning to conceive were found to be highly influential on the dynamic of social control. Wives reported making additional smoking rules or becoming stricter in enforcing the rules once they planned to conceive, became pregnant or the child was born. Some wives also gave child-related reasons when asking their husbands to stop or reduce their smoking, for example by telling their husbands that the money used for smoking could be used for the child’s needs. Some wives also implied that his smoking would hinder their chance of conceiving:

*So yes that’s probably my weapon. If it’s about a child, maybe [he would quit smoking]. ‘Don’t smoke! So we could have a child soon!’* (Arum, wife).

However, some wives were more lenient in enforcing the smoking rules after they had children. They said that, as long their husbands did not smoke near them or the children, they did not care as much.

Parenthood also affected the smokers, as they reported changing their smoking behaviour with or without their wives’ requests. This change varied from smoking less and paying more attention to the harm that second-hand smoke could cause the child to lecturing other smokers who smoked near children. Protecting their child was the common ground between wives and smokers:

*I’m sure if someone has a family um it then influences their ways of thinking, be it the children, or the financial need, they all change right. … For example then [before I had a child] I could smoke whenever I wanted. Well now if I want to [smoke] when I’m with my children, of course, I’ll wait. There’s no way I would smoke in front of my children.* (Teguh, smoker).

However, not all smokers could be swayed with child-related reasons, as the smokers or their wives often had parents who smoked, who the smokers would use as proof that it is possible to be both a parent and a smoker:

*I said [to husband] ‘maybe you should cut back [on smoking]’. [Husband] then said ‘my father smoked, yours as well. Heavily. [They] still have children anyway.’ (laughs) What am I supposed to say? Especially because my mother also used to smoke. And it was all fine, so there is no problem[,] right?* (Nisa, wife).

The health of other family members was also given as a reason for smokers to change their smoking behaviour. For example, one smoker said that he had started smoking less and had banned his siblings and father from smoking inside the house because his wife was allergic to it. Another started paying more attention to the danger of second-hand smoking after a doctor told him that his wife’s amniotic fluid was of an abnormal colour due to his smoking. The smokers did not report any significant change in their smoking behaviour when they were the ones who fell ill. However, it is important to note that the study criteria excluded participants with smoking-related chronic illnesses.

## Discussion

This study examined the topic of spousal social control in Indonesian single-smoker couples. Our findings provide insight into how wives feel about smoking, their attempts to encourage their husbands to cut back on or stop smoking, and how the smokers react to this. All the wives expressed a desire for their husbands to stop smoking, although only a few insisted or acted on this desire. The wives were able to control their husbands’ smoking to a certain extent, for example by limiting the places where they could smoke, but they were unable to make them stop. The smokers reacted positively to and were accepting of their wives’ smoking rules, but other tactics such as demanding that the smoker stop fell on deaf ears or induced conflict. Parenthood was a turning point for both wives and smokers: it was the time not only when wives put more pressure on their husbands to reduce or relocate their smoking but also when the smokers actively tried to change their smoking behaviour with or without their wives’ insistence.

Despite their generally negative attitudes toward smoking, the wives in this study tolerated it to a certain extent. Most wives grew up with at least one smoker in the family, and one had previously been a social smoker herself. Considering the high number of smokers in Indonesia, it is likely that the wives are conditioned to view smoking as normal, even if they do not like it. Bottorff et al.’s study, which examined women’s perspectives of their husbands’ continued smoking during their pregnancy and the postpartum period [40], considered the wives’ attitudes to their husbands’ smoking as representing ambivalent femininity. The wives complied and co-operated in ways that accommodate the masculine attributes of smoking (i.e. considering smoking to be a right or ensuring that the smokers eat healthily and exercise to counter the adverse effects of smoking) but also resisted it through social control [40].

The wives in this study favoured positive, direct social control tactics such as creating a pleasant situation using humour and negotiation. This is in line with the results of Lewis et al., who found using humour and making structural changes, such as limiting spaces to smoke, to be more successful than other tactics [34]. Despite a clear preference for non-confrontational tactics, most wives employed a combination of both confrontational and non-confrontational tactics, depending on the circumstances. Wives’ occasional confrontational, negative tactics seemed to be met only with negative behavioural reactions, i.e. ignoring, lying or hiding their smoking. The smokers did not seem to exhibit negative affect, nor did it significantly affect their relationship satisfaction, as they reported they rarely had fights with their wives about smoking. This is partially in line with Craddock et al.’s meta-analysis about the effectiveness of social control in changing health behaviours, showing that confrontational, negative tactics were often met with rejection from the smokers or even caused a fight [31]. However, Craddock et al. also found that social control targets reported negative affect as a result of receiving negative social control [31], which was not the case in this study. It is possible that the smokers did not report negative affect since the negative tactics were only used occasionally, and the wives carefully chose when to use certain tactics. Alternatively, cultural influences may play a role in the absence of negative affect in smokers whose wives used negative social control, which we will discuss further in the next paragraph. In general, the positive, non-confrontational tactics were accepted and produced better results than negative, confrontational tactics. Our findings are in line with previous social control studies [31], however, we found that social control is not enough to achieve smoking cessation.

The wives also considered their positions as wives, their husbands’ feelings, and the harmony of their relationship when exerting social control. The popularity of negotiation and non-confrontational tactics among the wives might be due to the idea that—as wives—they should consider their husbands’ feelings, but it could also be due to the existing communication pattern in Indonesia that prefers avoiding confrontation [41]. Most of the couples in this study were Javanese, the biggest ethnic group in Indonesia, and Javanese social and cultural practices heavily involve smoking [22]. Their ideology stems from peace, and they value harmony as the ultimate goal in life [42]. The Javanese also have strong patriarchal values [43], placing women in a perpetually lower position than men [43, 44]. The ideology and values might explain both the wives’ reluctance and leniency toward smoking and both partners’ preference for maintaining harmony.

The wives’ behaviours and the factors that they took into consideration, such as their position as wives and maintaining harmony, were very similar to how Chinese women manage smoking in their household [37, 38]. These similarities suggest that these factors might be essential in implementing spousal social control in patriarchal societies. Although the wives only felt limited power, these findings differ from those of Nichter et al.’s study [23], in which the participants reported lacking the ability to enforce smoking rules, which the wives in the current study were able to do.

Smokers accepted their wives’ smoking rules because of the role that the wives have as guardian of the family members’ health. These findings contrast with those of Nichter et al. [5], who found that smokers would consider their wives to be disrespectful if the wives tried to influence their smoking habits. We found that wives could regulate smoking to a certain extent; however, resistance did occur if the wives tried tactics that threatened the smokers’ autonomy to smoke, such as demanding that they stop altogether. This would result in lies and pretence from the smokers and the feeling of being pressured. This is similar to Kwon et al.’s [45] findings, in which smokers reported only reacting positively to spousal support—for example in the form of faith that the smokers would eventually stop—and if smokers were given complete autonomy in deciding when to stop. Anything other than support, such as pressure to stop, was perceived as challenging the smokers’ independence, freedom and, inadvertently, masculinity.

Although it has long been known that being male is the strongest predictor of smoking [46], the concept that smoking is related explicitly to gender as a social construct has only been studied in the last few decades, with a peak between 2007 and 2016 [17]. Masculinity constructs or the resources that boys and men utilise to prove their masculinity have been demonstrated to be harmful to their positive health behaviours [47, 48]. For example, behaviours such as not caring for their health or being involved in high-risk practices (e.g. reckless driving or substance abuse) are some of the ways of proving hegemonic masculinity ideals of being strong, tough and embracing risk [47–50]. Similarly, all components of smoking—initiation, continuation and stopping—and the ways in which wives could influence these are very likely to be affected by masculinity constructs.

Parenthood afforded the wives more power in controlling their husbands’ smoking, as both wives and smokers unanimously agreed that protecting their child, or their chance to have a child, was their top priority. Wives were found to enforce stricter or additional smoking rules after parenthood, and the smokers would abide by any child-related rules. Parenthood also evoked a shift in the smokers’ point of view, as having a child was mentioned as the moment at which they changed or decided to change their smoking behaviour. Various studies among male smokers have shown that a shift in smoking behaviour following recent fatherhood is common [51–53], especially in men who are involved in childcare [52, 54]. This shift has previously been explained as the result of adapting masculine ideals to the new roles of parenthood, such as protectors of the family and providers [40, 55].

To our knowledge, this is the first study to examine spousal social control in Indonesian smokers. Our findings could serve as a starting point for future studies into the social aspects of smoking, especially concerning romantic relationships. There are a few limitations to consider when interpreting the results of the present study. Firstly, the participants were recruited with the help of the first author’s social circle. While the first author and the second interviewer did not personally know any of the participants, the participants had some similar characteristics to the first author, such as socioeconomic status and education level. Secondly, most of the smokers indicated an intention to stop smoking in the future, so that the experiences of smokers who were further away from the behaviour change were not represented. Lastly, we did not have any information about the wives’ smoking histories. One of the wives mentioned that she used to be a social smoker, but the others did not mention any history of smoking. However, we did not specifically ask about this and therefore did not report it in the results.

Future research might consider studying further how parenthood affects smoking in Indonesia. In this study, the smokers who were yet to have a child also reported an intention to change their smoking behaviour for their future child. This suggests that parenthood might be a relevant factor for married smokers in general. Studies in Western smokers support the idea that parenthood is associated with less smoking [51, 55]. However, how parenthood affects Indonesian and Asian smokers remains unknown.

In conclusion, this study found that wives do exert social control and smokers are willing to accommodate them and adapt their smoking. However, wives may have a limited influence on smoking in Indonesia. Those who want to exert power might want to consider focusing on managing their husbands’ smoking at home instead of overall smoking. House rules and involving their husbands more in parenting could be an effective means of doing so.

## Electronic supplementary material

Below is the link to the electronic supplementary material.

Supplementary file1 (DOCX 16.8 kb)
