# Supplementary material for: Smoking-Related Social Control in Indonesian Single-Smoker Couples
Source: Int J Behav Med. 2020 Nov 10;28(4):455–65. doi: 10.1007/s12529-020-09935-z (PMC8263448; doi:10.1007/s12529-020-09935-z)
Supplement: Supplementary file 1 — Supplementary file1 (DOCX 16.8 kb) [file 12529_2020_9935_MOESM1_ESM.docx]

1. Smoker

| **Theme** | **Topic** | **Interview questions** |
| --- | --- | --- |
| Background | Daily life | Age  Education  Occupation  Relationship duration  Number of family member living in the same household (kids, parents, in-laws, does anyone else smoke?) |
| Smoking habit | Smoker identity, smoking habit | Could you tell what your daily activity related to smoking / daily smoking habit is like?  How is your smoking habit from when you first started until now? What is your plan for the future? |
| Spouse’s attitude | Spouse’s attitude toward smoking | How did your wife feel about your smoking habit before you were married?  How about now? Could you tell how your wife usually reacts to your smoking habit?  How does your wife’s reaction influence your smoking habit? |
| Social control | Spouse’s attempt to change participant’s smoking habit | How does your wife try to make you quit smoking?  How do you think a wife could make her husband quit smoking? Why do you think so?  What do you think is the most effective way to make a smoker quit?  What do you think is the most effective way to make you quit smoking? |

2. Non-smoking spouse

| **Theme** | **Topic** | **Interview questions** |
| --- | --- | --- |
| Background | Daily life | Age  Education  Occupation  Relationship duration  Number of family member living in the same household (kids, parents, in-laws, does anyone else smoke?) |
| Spouse’s smoking | Attitude toward spouse’s smoking | What do you think about smoking? How do you feel about it?  How did you feel about your husband’s smoking before you were married?  How about now?  Could you tell what your husband’s smoking habit is like?  Could you tell how you react when your husband smokes? How does your husband react, emotionally and in his smoking behavior? |
| Social control | Attempt to change spouse’s smoking habit | How do you try to make your husband quit or smoke less?  How do you think a wife can make her husband quit smoking? Why do you think so?  What do you think an effective way to make a smoker quit? |
